# Successful management of refractory chylous ascites using a Denver shunt following conversion surgery for unresectable locally advanced pancreatic cancer

**DOI:** 10.1007/s12328-026-02376-3

**Published:** 2026-06-16

**Authors:** Dongha Lee, Keiko Kamei, Masaya Nakano, Katsuya Ami, Chihoko Nobori, Yuta Yoshida, Takaaki Murase, Kentaro Tai, Atsushi Takebe, Ippei Matsumoto

**Affiliations:** Department of Surgery, Faculty of Medicine, Kindai University, 1-14-1 Mihara-dai, Minami-ku, Sakai, Osaka 590-0197 Japan

**Keywords:** Denver shunt, Refractory chylous ascites, Conversion surgery

## Abstract

We describe a case of refractory chylous ascites (RCA) successfully managed with a Denver shunt (DS) after conversion surgery for unresectable locally advanced pancreatic cancer. A 55-year-old woman developed RCA 6 months after distal pancreatectomy with celiac axis and portal vein resection. Conservative therapy, lymphangiography, and repeated paracentesis were ineffective. After confirming negative cytology, culture, and endotoxin tests, DS placement on postoperative day 425 resulted in rapid resolution of ascites, accompanied by improvement in body weight and hypoalbuminemia. No early major complications were observed after DS placement, and she was discharged safely. Although the shunt was removed 29 months later because of internal jugular vein thrombosis, no reaccumulation of ascites was observed thereafter. The patient remains alive without reaccumulation of ascites or tumor recurrence 60 months after curative resection. Although careful patient selection is essential because of potential complications, DS may serve as an effective salvage option for postoperative RCA when conservative approaches fail.

**Supplementary Information:**

The online version contains supplementary material available at https://doi.org/10.1007/s12328-026-02376-3.

## Introduction

Refractory chylous ascites (RCA) is a serious postoperative complication that leads to deterioration in nutritional status and prolonged hospitalization. In patients undergoing resection for pancreatic cancer, the incidence of chylous ascites has been reported to approximately 10% [1]. Most cases of chylous ascites improve with conservative management, including a medium-chain triglyceride diet [2, 3] total parenteral nutrition [4], and somatostatin analogs such as octreotide [5]. However, some patients do not respond adequately and develop RCA. RCA can be difficult to manage and may severely impair nutritional status and quality of life. As a result, it may delay or preclude the initiation of postoperative adjuvant chemotherapy, which could negatively impact long-term oncological outcomes [6].

In addition to conservative therapy, treatment options for RCA include lymphangiography with or without embolization [7] and, in selected cases, surgical ligation of the leaking lymphatic vessels. However, these procedures are technically challenging and invasive, particularly in patients with poor nutritional status or comorbidities. A peritoneovenous shunt, such as the Denver shunt (DS), offers an alternative option for controlling RCA by redirecting ascitic fluid into the systemic circulation via a pressure gradient between the peritoneal cavity and central veins. DS is typically used for palliative purposes in patients with malignant ascites or hepatic ascites due to decompensated cirrhosis [8, 9]. However, its indication following potentially curative surgery remains unclear because of concerns regarding disseminated intravascular coagulation (DIC), infection, heart failure, and shunt occlusion.

Here, we report a case of successfully managed RCA with DS following curative resection for unresectable locally advanced pancreatic cancer. This case highlights the potential role of DS as a salvage treatment even in patients with favorable prognosis when conservative and interventional treatments fail.

## Case report

**Fig. 1:**
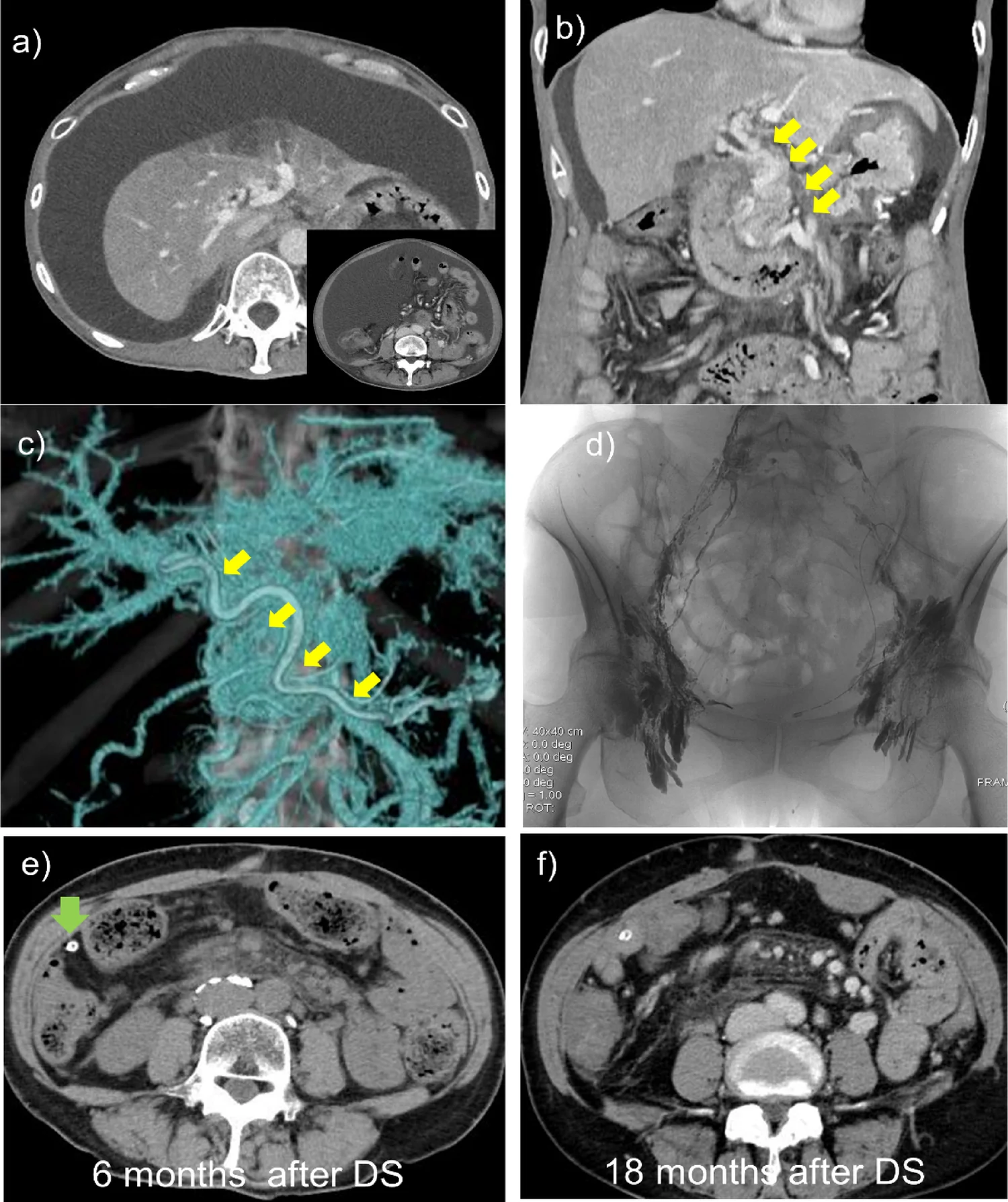
Imaging findings and postoperative course. **a** Computed tomography performed 6 months after surgery showed massive ascites. **b** Coronal CT and **c** three-dimensional reconstruction images demonstrated development of collateral vessels around the hepatoduodenal ligament and common bile duct due to portal vein obstruction. Yellow arrows indicate the area of portal vein obstruction. **d** Intranodal lymphangiography failed to identify a definite lymphatic leakage site. **e**, **f** Follow-up CT performed 6 and 18 months after Denver shunt placement showed complete resolution of ascites without reaccumulation. The green arrow indicates the Denver shunt catheter

A 55-year-old woman was diagnosed with unresectable locally advanced pancreatic cancer located in the pancreatic body and tail. The approximately 5-cm tumor circumferentially involved the splenic artery and the root of the common hepatic artery, contacted more than half of the superior mesenteric artery circumference, and encased more than 180° of the superior mesenteric vein at the portal vein confluence. From October 2020 to March 2021, she received four courses of gemcitabine plus nab-paclitaxel followed by preoperative S-1-based chemoradiotherapy (50 Gy in 25 fractions). After treatment, computed tomography (CT) showed tumor shrinkage from approximately 5–3 cm, and the serum CA19-9 level decreased from 1822 to 87 U/mL. The radiological response was assessed as partial response according to the Response Evaluation Criteria in Solid Tumors version 1.1 [10]. After a favorable response, she underwent distal pancreatectomy with celiac axis resection and portal vein resection with reconstruction. Pathological examination of the resected specimen revealed invasive ductal adenocarcinoma of the pancreatic body and tail, measuring 60 mm in diameter. The pathological stage was pT3N1aM0, corresponding to stage IIB according to the 8th edition of the UICC classification [11]. R0 resection was achieved, with no lymphatic or venous invasion (ly0, v0), and the histological response to preoperative therapy was classified as Evans grade III. She was discharged on postoperative day (POD) 30 without major complications. However, approximately six months after surgery, she gradually developed abdominal distension, and CT confirmed massive ascites with stenosis and partial obstruction at the portal vein reconstruction site and collateral vessels around the hepatoduodenal ligament and common bile duct (Fig. 1a–c). Diagnostic paracentesis yielded milky fluid, and the ascitic triglyceride level was 504 mg/dL, confirming chylous ascites.

**Fig. 2:**
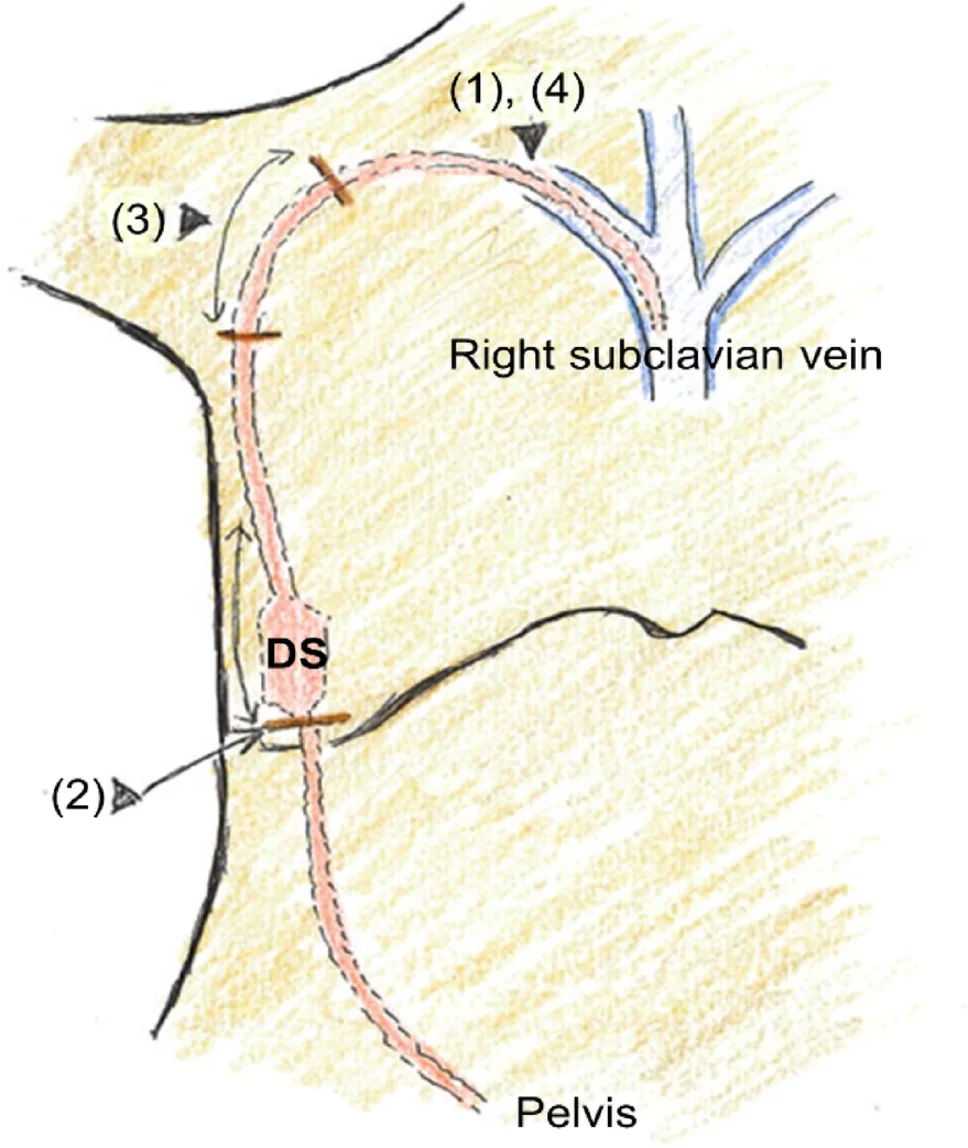
Procedure for Denver shunt (DS) placement. (1) ultrasound-guided puncture of the right subclavian vein followed by insertion of a guidewire; (2) a 3-cm skin incision and creation of a subcutaneous pocket just beneath the right costal arch, followed by ultrasound-guided peritoneal puncture with placement of the peritoneal end of the catheter into the pelvic cavity; (3) creation of a subcutaneous tunnel directed toward the right subclavian vein puncture site; (4) advancement of the venous limb of the catheter over the guidewire into the right subclavian vein

**Fig. 3:**
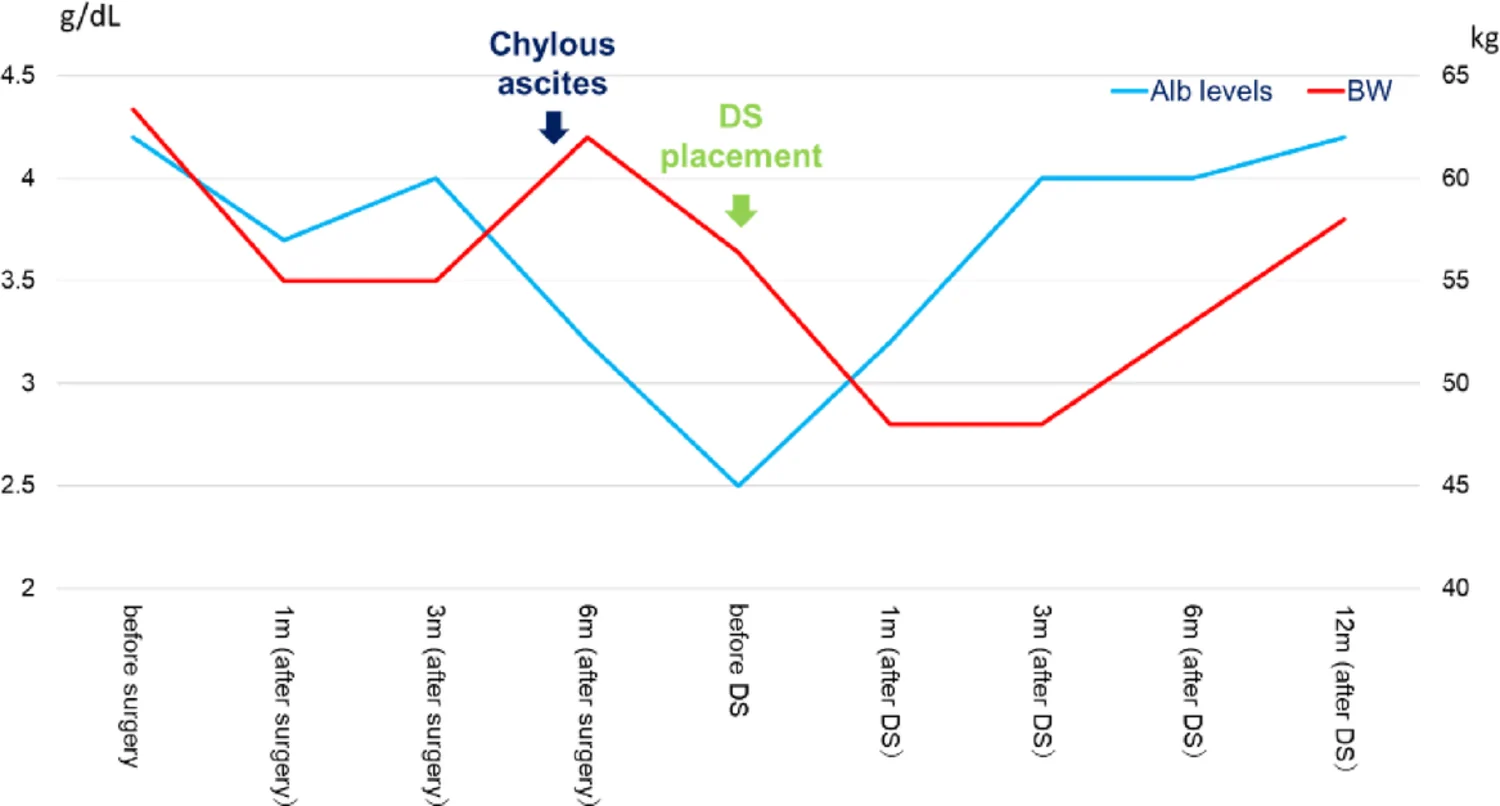
Changes in body weight and serum albumin levels before and after Denver shunt (DS) placement. Body weight decreased from 56.4 kg before placement to 48.0 kg at 1 month and remained stable thereafter. Serum albumin increased from 2.5 g/dL pre-placement to 3.2 g/dL at 1 month and 4.0 g/dL at 3 months after DS placement BW: body weight, Alb: albumin

Conservative management—including a low-fat diet, diuretics, and octreotide—was ineffective. Lymphangiography failed to identify the leaking lymphatic vessel (Fig. 1d), and repeated paracenteses and sessions of cell-free and concentrated ascites reinfusion therapy (KM-CART) did not improve the ascites. Prior to shunt placement, ascitic cytology, culture, and endotoxin tests were all negative. On POD 425, DS was placed under general anesthesia. The operative time was 91 min, and the procedure consisted of the following steps: (1) ultrasound-guided puncture of the right subclavian vein followed by insertion of a guidewire; (2) a 3-cm skin incision and creation of a subcutaneous pocket just beneath the right costal arch, followed by ultrasound-guided peritoneal puncture with placement of the peritoneal end of the catheter into the pelvic cavity; (3) creation of a subcutaneous tunnel directed toward the right subclavian vein puncture site; (4) advancement of the venous limb of the catheter over the guidewire into the right subclavian vein (Fig. 2). After DS placement, the ascites resolved rapidly. Her abdominal distension and bilateral lower extremity edema markedly improved. Figure 3 shows changes in body weight and serum albumin levels. Her body weight decreased from 56.4 to 48.0 kg within one month and remained stable. Serum albumin levels increased from 2.5 g/dL before DS placement to 3.2 g/dL at one month, and 4.0 g/dL at three months postoperatively, reflecting improved nutritional status. As shown in Fig. 1e, f, follow-up CT after DS placement demonstrated complete resolution of ascites. Although the shunt was removed 29 months after DS placement because of internal jugular vein thrombosis, no reaccumulation of ascites was observed thereafter. Serial follow-up CT showed no evidence of local recurrence, distant metastasis, or peritoneal dissemination. Serum CA19-9 levels decreased after preoperative treatment and subsequently remained low without re-elevation during follow-up. The patient remains alive without reaccumulation of ascites or tumor recurrence 60 months after curative resection.

## Discussion

Chylous ascites is a known complication after gastrointestinal surgery, resulting from intraoperative injury to the lymphatic system with subsequent lymphatic leakage into the peritoneal cavity [12]. Advanced pancreatic cancer is frequently characterized by extensive neural plexus invasion, lymph node metastasis, and lymphatic vessel involvement, which necessitate aggressive surgical approaches with extensive lymphadenectomy [13]. Consequently, chylous ascites has been reported to occur in approximately 1–16% of patients undergoing pancreatic resection [1, 12, 14]. Although most cases are recognized in the early postoperative period, delayed-onset chylous ascites several weeks to months after pancreatic surgery has also been reported and may be related to lymphocele formation and subsequent slow leakage of chyle [15]. In the present case, such a delayed mechanism may have been compounded by preoperative chemoradiotherapy-induced fibrosis, extensive lymphatic dissection, and progressive portal venous flow disturbance after portal vein reconstruction. In recent years, conversion surgery for unresectable locally advanced pancreatic cancer has been increasingly performed in selected patients who respond favorably to intensive chemotherapy and chemoradiotherapy [16]. However, conversion surgery is generally more technically demanding and aggressive than standard pancreatic resection, often involving locally advanced bulky tumors and radiation- or chemotherapy-induced fibrosis following preoperative treatment, and frequently requiring wider dissection as well as vascular resection or reconstruction. As a result, conversion surgery is associated with a higher risk of postoperative complications, including lymphatic leakage and chylous ascites, compared with standard pancreatic resection [17]. In the present case, conversion surgery with portal vein resection and reconstruction following preoperative chemoradiotherapy was performed, and despite conservative management, RCA subsequently developed. RCA causes nutritional deterioration and interferes with postoperative management, often delaying or preventing adjuvant chemotherapy. Previous reports have shown that the 3-year survival rate was 18.8% in patients with uncontrolled postoperative chylous ascites, compared with 53.4% in those with successful ascites control after pancreatic cancer surgery [6]. Therefore, effective management of RCA is essential to allow recovery and continuation of oncological treatment.

When conservative therapy fails to control postoperative chylous ascites, alternative treatment options include lymphangiography or surgical ligation of leaking lymphatic vessels; however, both approaches are often limited by technical difficulty and invasiveness in the postoperative setting. Portal vein stenting may also be considered when ascites is associated with postoperative portal vein stenosis or occlusion. In the present case, however, the ascites was clearly chylous, with a triglyceride level of 504 mg/dL, suggesting that lymphatic leakage, rather than portal hypertensive ascites, was the major contributing factor. The ascites remained refractory despite conservative and symptomatic treatments, including lymphangiography, repeated paracentesis, and KM-CART. At the time DS was considered, the portal vein had already progressed to long-segment chronic occlusion, and hepatopetal collateral pathways were well developed. In addition, according to the interventional radiology assessment, percutaneous transhepatic intervention was considered less suitable in the presence of massive ascites, and a transileocolic approach under laparotomy and general anesthesia would likely have been required. Therefore, both the technical feasibility and the additional clinical benefit of portal vein stenting were considered uncertain. Since no large comparative studies have established superiority between portal vein stenting and DS placement for refractory chylous ascites, the optimal strategy remains uncertain and treatment should be individualized according to the predominant pathophysiology and technical feasibility [18]. For these reasons, DS was prioritized as the more feasible and less invasive salvage option in this setting. DS has been reported to provide rapid symptomatic relief, with clinical response rates of 92.3% in patients with chylous ascites and approximately 85% in those with malignant ascites, indicating high immediate effectiveness after placement [19, 20]. In the present case, DS placement resulted in prompt improvement of abdominal symptoms. Additionally, nutritional status improved markedly, with sustained long-term control of ascites. Although the shunt was removed 29 months after DS placement because of internal jugular vein thrombosis, no reaccumulation of ascites occurred thereafter, and the patient remained free of both ascites and tumor recurrence 60 months after curative resection.

However, DS placement is associated with potentially serious complications. Among these, DIC is the most critical, reported in 2–10% of cases and typically occurring within the first few days after placement due to activation of the coagulation cascade by rapid reinfusion of ascitic fluid [21, 22]. In addition, catheter-related infection and the theoretical risk of hematogenous tumor dissemination remain important concerns, particularly in the postoperative setting of pancreatic cancer [23, 24]. Therefore, careful patient selection is essential when considering DS placement. In the present case, these risks were minimized through strict patient selection and perioperative management. Before DS placement, the absence of malignancy and infection was confirmed by negative ascitic cytology, culture, and endotoxin tests. After DS placement, the patient was closely monitored postoperatively, and no early major complications, including DIC, infection, or heart failure, were observed. Although cytology of ascitic fluid was negative before DS placement in the present case, occult malignancy could not be completely excluded. Therefore, the indication for DS placement after surgery for malignant disease remains controversial and should be determined cautiously. Previously reported cases of DS placement for refractory postoperative ascites after surgery for malignant diseases are summarized in Table 1. In these reports, DS was selected after failure of conservative treatment and/or lymphangiography, and cytology before DS placement was negative in all cases. In the present case, R0 resection was achieved, ascitic cytology before DS placement was negative, and no radiological evidence of recurrence or peritoneal dissemination was observed at the time of DS placement. These findings supported the use of DS as an acceptable salvage option. However, careful long-term follow-up is mandatory because the oncological safety of DS after curative-intent surgery for pancreatic cancer remains unclear.

**Table 1:** Previously reported cases of Denver shunt placement for refractory postoperative ascites after surgery for malignant diseases

No.	Author, year	Age	Primary disease	Surgery	Type of ascites	Previous treatment	Cytology before DS	Timing of DS	Outcome	Recurrence after DS
1	Inoue et al. [25], 2011	72	Hepatocellular carcinoma	Partial hepatectomy	Non-chylous lymphatic ascites	Albumin/plasma protein infusion, diuretics	Negative	POD 98	Ascites resolved; nutritional status improved	No ascites or tumor recurrence at 20 months
2	Sakamoto et al. [26], 2022	74	Gastric cancer	Total gastrectomy with para-aortic lymph node dissection	Chylous ascites	Dietary therapy, diuretics, octreotide, lymphangiography, CART	Negative	Approximately 4 months after surgery	Ascites resolved; nutritional status improved	No ascites or tumor recurrence at 13 months
3	Nakajima et al. [27], 2025	80	Cholangiocarcinoma	Extended right hemihepatectomy with lymphadenectomy	Chylous ascites	Dietary therapy, TPN, diuretics, octreotide, lymphangiography	Negative	Approximately 4 months after surgery	Ascites decreased; general condition improved	Liver metastasis at 10 months
4	Present report, 2026	55	Pancreatic cancer	Distal pancreatectomy with celiac axis resection and portal vein resection	Chylous ascites	Dietary therapy, diuretics, octreotide, lymphangiography, KM-CART	Negative	POD 425	Ascites resolved; nutritional status improved	No ascites or tumor recurrence at 60 months

CART, cell-free and concentrated ascites reinfusion therapy; DS, Denver shunt; KM-CART, Kansai Medical University cell-free and concentrated ascites reinfusion therapy; POD, postoperative day; TPN, total parenteral nutrition

DS placement for refractory ascites has traditionally been used as a palliative treatment, mainly in patients with malignant ascites or liver cirrhosis [8, 9]. As shown in Table 1, only a limited number of reports have described DS placement for refractory postoperative ascites after surgery for malignant diseases [25–27]. To our knowledge, however, no reports have described DS placement after curative-intent resection for pancreatic cancer. These findings suggest that DS can provide meaningful clinical benefit when used selectively in postoperative pancreatic cancer patients with refractory ascites. Nevertheless, DS placement should be considered only after thorough evaluation and when other therapeutic options have failed. Careful patient selection, close postoperative monitoring, and long-term oncological follow-up are essential.

We report a case of RCA successfully managed with DS after conversion surgery for unresectable locally advanced pancreatic cancer. DS placement can be an effective salvage option for postoperative RCA when conservative and interventional treatments fail. With careful patient selection and exclusion of infection or malignant ascites, this strategy may facilitate clinical recovery even in patients with potentially favorable prognosis.

## Supplementary Information

Below is the link to the electronic supplementary material.

Supplementary Material 1

## Data Availability

Not applicable.
